# Autonomic cardiovascular control in older patients with acute infection and delirium: a pilot study of orthostatic stress responses

**DOI:** 10.1186/s12877-019-1035-0

**Published:** 2019-01-25

**Authors:** Bjørn Erik Neerland, Torgeir Bruun Wyller, Vegard Bruun Bratholm Wyller

**Affiliations:** 1 Department of Geriatric Medicine, Oslo University Hospital, Oslo, Norway; 2 Institute of Clinical Medicine, University of Oslo, Oslo, Norway; 3 Department of Pediatrics, Akershus University Hospital, Lørenskog, Norway; 4 Oslo Delirium Research Group, Department of Geriatric Medicine, University of Oslo, P.O.BOX 4956, Nydalen, N-0424 Oslo, Norway

**Keywords:** Delirium, Head-up tilt, Autonomic function, Heart rate variability

## Abstract

**Background:**

Alterations in autonomic nervous system (ANS) activity might be involved in the pathophysiology of delirium. The aim was to explore autonomic cardiovascular control in older patients with and without delirium.

**Methods:**

Fourteen patients (five with delirium) acutely admitted to the geriatric ward with an infection were enrolled in the study. Patients with atrial fibrillation, a pacemaker, or on treatment with beta-blockers, calcium channel blockers or acetylcholinesterase inhibitors were not eligible.

Continuous, non-invasive hemodynamic variables were measured during supine rest (5 min) and head-up tilt (HUT) to 15 degrees (10 min). Heart rate (HR), blood pressure (BP) and stroke volume (SV) were recorded beat-to-beat. Cardiac output (CO), total peripheral resistance (TPR), end-diastolic volume (EDV) and heart rate variability (HRV) values were calculated.

**Results:**

Median age was 86 years. HR, BP, SV, CO, TPR and EDV were similar across the two groups at rest, but there was a trend towards a greater increase in systolic BP and HR during HUT in the delirium group. At rest, all HRV indices were higher in the delirium group, but the differences were not statistically significant. During HUT, the delirium group had higher power spectral density (PSD) (representing total variability) (*p* = 0.06) and a lower low frequency (LF)/high frequency (HF)-ratio (an index of sympathovagal balance) than the control group (*p* = 0.06). Also, delirious patients had a significantly greater reduction in standard deviation of RR-intervals (SDNN) (representing total variability) from baseline than controls (*p* = 0.01) during HUT.

**Conclusions:**

This explorative pilot study on autonomic cardiovascular control in delirium suggests that there may be differences in HRV that should be further investigated in larger samples.

## Background

Delirium is a common disorder among hospitalised older patients, characterised by an acute onset and fluctuating course, altered arousal, inattention and cognitive problems [1]. Infections are among the most common precipitating causes of delirium. Despite its severity, the underlying pathophysiology of delirium is poorly understood [2]. The clinical features of delirium might suggest aberrations in acute stress responses, involving alterations in autonomic nervous system (ANS) activity [3].

ANS activity can be indirectly assessed by measuring heart rate variability (HRV). HRV refers to the beat-to-beat variations in the sinus rhythm and results from the constant interaction between sympathetic and parasympathetic activity [4]. When subjected to an orthostatic challenge (e.g., head-up tilt testing [HUT]), the ANS must compensate to maintain cardiovascular homeostasis.

Overall HRV decreases with age [5], is negatively associated with frailty status [6], and is inversely related to infection biomarkers such as C-reactive protein (CRP) [7, 8]. Further, there is evidence that reduced HRV is associated with cognitive impairment [9–11]. HRV in patients with delirium has been investigated previously in two studies, both in intensive care unit (ICU) patients [12, 13]. Zaal and co-workers did not find any significant differences in HRV parameters between ICU patients with (*n* = 12) and without (*n* = 13) delirium [12], whereas a recent study (*n* = 60) from South Korea found an association between delirium and increased HRV [13]. To our knowledge, HRV in delirium has not previously been studied outside the ICU, and especially not in older medical patients.

The aim of our study was to explore autonomic cardiovascular control in older medical patients with and without delirium, and particularly to assess possible differences in HRV according to delirium status. Due to the lack of previous knowledge about ANS activity during an episode of delirium, an explorative approach was chosen.

## Methods

### Patients

Patients admitted acutely with an infection to the acute geriatric ward at Oslo University Hospital were eligible for the study. We excluded patients with atrial fibrillation, pacemaker rhythm, or on current treatment with beta-blockers, calcium channel blockers or acetylcholinesterase inhibitors. The recruitment was pragmatic and took place whenever the investigator had the opportunity to be in the ward. If the patients did not fulfil any exclusion criteria, they were asked by the investigator (BEN) to participate in a head-up tilt-test (HUT) the next morning, between 9 and 10 a.m., performed by the same investigator. Written and oral information was given by BEN to the patient and to their relatives. Written consent was obtained the next day.

### Ethics

The study was approved by the Regional Committee for Ethics in Medical Research in Norway (REK 2011/2498) on 28 February 2012, and by the Data Protection Authorities. The study was undertaken in accordance with the Declaration of Helsinki. Patients were enrolled based either on written consent or on presumed consent in combination with written assent from the nearest relative.

### Delirium assessments

Delirium was diagnosed by the researcher (BEN; physician trained in geriatrics) performing the HUT. The researcher visited the patient the day before HUT and spent time with the patient the next day. A structured patient interview, cognitive tests (Mini Mental State Examination [MMSE]) [14] and attention tests (digit span) were performed shortly after HUT. The Confusion Assessment Method (CAM) [15] was used for delirium screening, and “Months Of The Year Backwards” [16] and “Days Of The Week Backwards”, as well as DelApp [17] and the Observational Scale of Level of Arousal (OSLA) [18] were also used in the delirium assessment. All chart notes (nurses and doctors) were scrutinized for evidence of delirium symptoms during night-shifts, possible fluctuations, and resolution of symptoms. The delirium diagnosis was made according to the DSM-5 criteria [19], based on a comprehensive approach; observing and interviewing the patient, test results, and information from staff members and case notes. Delirium severity was assessed using the Memorial Delirium Assessment Scale (MDAS) [20].

### Head-up tilt tests (HUT)

All HUTs were performed in the morning, between 9 and 10 a.m., in a quiet room with comfortable temperature, by the same investigator (BEN). The HUT took place a number of days after admission, to ensure that the patients were rehydrated and initial medical treatment had started. The patients had fasted overnight, but were allowed to drink (water, juice, milk) and to take their medicines. They were lying supine in their hospital bed, and were attached to a Task Force Monitor (TFM) (CNSystems Medizintechnik, Graz, Austria), a combined hardware and software device for noninvasive recording of cardiovascular variables. Baseline recordings were obtained for 5 min while the patients rested horizontally, before they were tilted head-up to 15 degrees for 10 min. The subjects were asked to relax and remain silent except for comments about discomfort. The low tilt angle chosen (15 degrees) is challenging for the cardiovascular system, but produces less discomfort than larger angles, and was the largest angle possible using the regular hospital beds.

### Recordings

Four electrodes were placed for ECG recording, and three band electrodes were placed on the neck and upper abdomen for impedance cardiography (ICG). ICG is a method in which a small electrical potential is applied between the band electrodes (placed on the neck and upper abdomen). This method gives a continuous recording of the transthoracic impedance (dZ/dt) [21]. Blood pressure (BP) was recorded oscillometrically and continuously using the Finapres finger plethysmography technique, recorded from the second or middle finger of the right hand [22]. The continuous BP was calibrated against the oscillometric measurement.

### Data analyses

All recordings were transferred online to the TFM for real-time data acquisition. Beat-to-beat stroke volume (SV) was calculated from the impedance signal, and beat-to-beat stroke index (SI) and cardiac index (CI) were obtained by dividing SV and cardiac output (CO) by body surface area (BSA), estimated from the subject’s height and weight. The total peripheral resistance index (TPRI) was calculated from the mean arterial blood pressure (MAP) divided by CO and BSA.

Two time series of 4 min were selected from each patient for analyses; 270–30 s prior to the 15^o^ HUT and 120–360 s after the tilt. Delta-tilt values were calculated (HUT - rest). Ectopic beats, artifacts and their corresponding BP values were manually removed and replaced by linear interpolation. We then computed the median of all cardiovascular variables in all time periods and calculated the medians with total range for the two groups (delirium/no delirium).

### Heart rate variability (HRV) analyses

The ECG signal was subjected to spectral analysis using an adaptive autoregressive algorithm that creates a time-varying spectrum [23] from consecutive segments of the biosignals [24, 25]. The length of these segments varies, as the biosignals are continuously monitored for departure from stationarity, and the segment boundaries placed accordingly. The power spectral density (PSD) is the power distribution across frequencies, representing total variability. For analysis of the power spectrum, we used fixed frequency bands, defined as: Very low frequency (VLF) < 0.04 Hz; low frequency (LF) 0.04–0.15 Hz, and high frequency (HF) 0.15–0.40 Hz. HF and LF power were converted to normalized units (nu); HFnu = HF/(total power - VLF)× 100. LF is considered to measure both parasympathetic and sympathetic modulation of the sine node; HF solely parasympathetic modulation, and the LF/HF-ratio to reflect the sympathovagal balance [4].

We also measured HRV using time domain analyses [4]. Standard deviation of RR-intervals (SDNN) is the standard deviation of all normal RR-distances and correlates with PSD, representing total variability. pNN50 is the proportion of successive RR-intervals that differ by more than 50 ms. RMSSD is the square root of the mean square differences of successive RR-intervals. pNN50 and RMSSD correlate with HF and are assumed to measure parasympathetic modulation.

### Background variables

Demographic data were collected at inclusion. Comorbidity was evaluated using the Cumulative Illness Rating Scale (CIRS) [26]. The American Society of Anesthesiologists (ASA) score [27] and the Acute Physiology and Chronic Health Evaluation version 2 (APACHE II) [28] score were used for assessment of physiological disruption. The proxy-based Informant Questionnaire on Cognitive Decline in the Elderly (IQCODE) [29] and the Clinical Dementia Rating scale (CDR) [30] were used to assess pre-existing cognitive impairment. Level of functioning was evaluated using the Barthel Activities of Daily Living Index [31], an index of primary activities of daily living. Venous blood samples were drawn and analysed, following standard hospital procedures. Body mass index (BMI) was calculated from height and weight. Weight was measured while standing or sitting on an electronic scale with light clothing, and height measured using a metric rule (patient either lying supine in bed or standing against a wall).

### Statistical analysis

The data were exported from the TFM software to Microsoft Excel for further preparation and then transferred to SPSS Statistics version 24 (IBM, Armonk NY) for statistical analysis. We computed the median of the hemodynamic variables in all the epochs and calculated the medians with total range for the groups separately. We applied parametric (t-test) and non-parametric (Mann-Whitney test) analyses for group comparisons as appropriate. The Wilcoxon signed rank test was used for comparisons of paired observations. A *p*-value of < 0.05 was considered statistically significant.

## Results

Fourteen participants (five with delirium) were included between March 2012 and January 2015 among patients admitted to the acute geriatric ward at Oslo University Hospital (Table 1). The patients had respiratory tract infections (*n* = 8), urinary tract infections (*n* = 4), erysipelas (*n* = 1) or cholecystitis (n = 1). Patients with delirium had higher CRP values at the time of HUT (median 174 [range 67–306] mg/L vs 62 [7–191] mg/L; *p* = 0.04). They were also older (median 89 [84–91] years vs 86 [74–91] years), had a higher CIRS score (median 14 [7–19] vs 10 [1–17]) and a lower Nottingham Extended Activities of Daily Living (NEADL) score (median 28 [9–30] vs 52 [13–66]), but these differences were not statistically significant. The percentage of patients with an IQCODE > 3.82 was similar in the delirium group (40%) and in the group without delirium (44%).

**Table 1:** Characteristics of participants according to delirium status

Characteristic	All Participants, *n* = 14	Delirium, *n* = 5	No Delirium, *n* = 9	*P*-value*
Age, median (range)	86 (74–91)	89 (84–91)	86 (74–89)	0.3
Women, n (%)	7 (50)	1 (20)	6 (67)	0.1
BMI, kg/m^2^, median (range)	23 (17–30)	21 (18–26)	23 (17–30)	0.7
Admitting diagnosis, n (%)				0.6
Pneumonia/Respiratory Tract Infection	8 (57)	4 (80)	4 (44)	
Urinary Tract Infection	4 (29)	1 (11)	3 (33)	
Erysipelas	1 (7)	0	1 (11)	
Cholecystitis	1 (7)	0	1 (11)	
Number of days from admission to HUT testing, median (range)	2 (1–5)	2 (2–2)	3 (1–5)	0.2
CRP maximum value, mg/L, median (range)	164 (24–347)	204 (70–306)	149 (24–347)	0.6
CRP value at HUT, mg/L, median (range)	110 (7–306)	174 (67–306)	62 (7–191)	0.04
Leukocytes, maximal count, cells*10^9^/L, median (range)	16 (5–24)	18 (10–20)	13 (5–24)	0.4
APACHE II score at HUT, median (range)	7 (6–10)	8 (7–10)	6 (6–10)	0.2
MDAS score, median (range)	8 (1–16)	16 (12–16)	4 (1–10)	0.001
Delirium subtype, n (%)				
Hypoactive delirium		4 (80)		
Hyperactive delirium		–		
Mixed-type delirium		1 (20)		
CIRS score, median (range)	12 (1–19)	14 (7–19)	10 (1–17)	0.1
IQCODE score, median (range)	3.7 (3.0–5.0)	3.6 (3.0–4.4)	3.7 (3.0–5.0)	0.7
IQCODE score > 3.82, n (%)	6 (43)	2 (40)	4 (44)	0.9
NEADL index score, median (range)		28 (9–30)	52 (13–66)	0.1
Barthel Index score, median (range)	17.5 (6–20)	17 (15–18)	19 (6–20)	0.3
Clinical Dementia Rating, median (range)	0.5 (0–2)	0.5 (0.5–2)	0.5 (0–2)	1.0
MMSE score, median (range)	21 (12–28)	15 (12–23)	21 (14–28)	0.04

*BMI* Body Mass Index, *IQCODE* Informant Questionnaire on Cognitive Decline in the Elderly, *CRP* C-reactive protein, *APACHE* Acute Physiology and Chronic Health Evaluation, *HUT* Head-up tilt test, *CIRS* Cumulative Illness Rating Scale, *CDR* Clinical Dementia Rating scale, *MMSE* Mini-Mental State Examination

*Chi-square tests and Mann-Whitney tests

### Autonomic response to HUT 15 degrees

Descriptive data for all patients during supine rest and HUT to 15 degrees show that HR and BP values were stable during tilt. There was a slight, but significant decrease in SI (*p* < 0.001) and an increase in TPRI (*p* < 0.001) during HUT (Table 2). Indices indicating increased sympathetic activity (LF-variables and LF/HF-ratio) also increased slightly, but these changes were not significant.

**Table 2:** Descriptive data for all patients (*n* = 14) during supine rest and HUT to 15° ^a^

Characteristic	Rest	15° tilt	Change after 15° tilt
Hemodynamic measures			
HR (beats/min)	66 (54–85)	68 (54–84)	0 (− 2–6)
SBP (mmHg)^b^	115 (88–136)	117 (86–132)	3 (− 8–29)
DBP (mmHg)^b^	67 (48–78)	70 (59–86)	1 (− 8–16)
MAP (mmHg)^b^	78 (58–96)	80 (66–104)	2 (− 8–22)
EDI	51 (37–72)	48 (38–66)	−4 (− 8–2)^†^
SI (ml/m^2^)	32 (23–42)	30 (23–39)	−3 (− 6–0)^†^
CI (l/min/m^2^)	2.3 (1.5–3.5)	2.0 (1.6 (3.2)	− 0.1 (− 0.4–0.1)^†^
TPRI (mmHg/l/min/m^2^)	11 (8–24)	14 (8–24)	2 (− 2–4)^†^
Frequency domain measures			
PSD RRI	444 (16–30,059)	514 (46–22,462)	−8 (− 7612–8015)
LFnuRRI	27 (11–61)	31 (9–65)	4 (− 28–16)
HFnuRRI	73 (39–89)	69 (35–91)	−4 (− 16–28)
LFabsRRI (ms^2^)	91 (5–11,813)	115 (10–11,579)	4 (− 234–860)
HFabsRRI (ms^2^)	158 (8–14,150)	233 (12–11,365)	3 (− 2784–7071)
LF/HF ratio	0.4 (0.1–1.6)	0.5 (0.1–1.9)	0.1 (− 0.7–0.8)
Time domain measures			
SDNN (ms)	28 (8–227)	28 (13–174)	−5 (− 54–16)
pNN50 (%)	3 (0–68)	3 (0–86)	0 (− 16–18)
RMSSD (ms)	22 (7–151)	30 (19–43)	7 (− 120–23)
BRS (ms/mmHg)	6 (3–53)	7 (3–55)	1 (− 10–31)

HUT = Head-up tilt test; HR = heart rate; SBP, DBP and MAP = systolic, diastolic and mean blood pressure respectively; SI = stroke index; CI = cardiac index and TPRI = total peripheral resistance index (Indexed values are stroke volume; cardiac output and total peripheral resistance divided by body surface area); EDI = End diastolic volume index; LFnuRRI and HFnuRRI = low frequency and high frequency power in normalized units; LF or HF/(Total Power-VFL(very low frequency) × 100; LFabsRRI and HFabsRRI = power in high frequency and low frequency range in absolute values, respectively; LF/HF = ratio low frequency power/high frequency power; SDNN = Standard Deviation of all NN intervals; pNN50 = NN50 count divided by the total number of all NN intervals; NN50 = number of interval differences of successive NN intervals greater than 50 ms; RMSSD = Root of the Mean of the Sum of Squares of Differences between adjacent NN intervals; NN intervals = normal-to-normal intervals; BRS = Baroreceptor reflex sensibility

^†^*p* < 0.001, Wilcoxon signed rank test

^a^Values are medians (total range) obtained from registration during sequences of four minutes at rest and tilt to 15°, and changes from rest after tilt to 15°

^b^BP-recordings are missing in 1 patient with no delirium

#### Hemodynamic regulation according to delirium status

At rest, values for HR, BP and indices for SV, CO, TPR and end diastolic volume were similar for patients with and without delirium (Table 3). There were also no significant differences between the groups in these conventional cardiovascular variables during HUT. However, there was a trend for both systolic BP and HR to increase more during tilt in the delirium group.

**Table 3:** Hemodynamic Regulation at Rest and During 15° HUT by diagnosis^a^

Characteristic	Rest		15° tilt		Change after 15° tilt	
	Delirium, *n* = 5	No delirium, *n* = 9	Delirium, *n* = 5	No delirium, *n* = 9	Delirium, *n* = 5	No delirium, *n* = 9
RR (breaths/min) median, range	20 (12–23)	17 (11–28)				
HR (beats/min)	68 (54–80)	66 (62–85)	70 (54–82)	66 (62–84)	2 (−1–3)	−1 (− 2–6)
SBP (mmHg)^b^	115 (99–136)	115 (88–126)	122 (103–131)	117(86–132)	7 (− 8–13)	−2 (− 7–29)
DBP (mmHg)^b^	69 (58–78)	65 (48–77)	70 (63–86)	68 (59–78)	4 (− 8–14)	1 (− 3–16)
MAP (mmHg)^b^	78 (68–96)	78 (58–92)	86 (72–104)	78 (66–92)	8 (− 8–14)	9 (− 3–22)
SI (ml/m^2^)	32 (23–33)	35 (28–42)	28 (23–31)	32 (26–39)	−3 (− 6–0)	− 4 (− 6 - -1)
CI (l/min/m^2^)	1.9 (1.5–2.6)	2.4 (1.8–3.5)	1.8 (1.6–2.4)	2.1 (1.6–3.2)	− 0.1 (− 0.4–0.1)	− 0.2 (− 0.4 - -0.1)
TPRI (mmHg/l/min/m^2^)	14 (10–17)	11 (7–23)	15 (11–21)	12 (8–24)	2 (−2–4)	1 (0–4)
BRS (ms/mmHg)	12 (6–36)	6 (3–53)	9 (5–55)	7 (3–43)	10 (− 1–31)	1 (− 10–10)
EDI (ml/m^2^)	50 (37–51)	55 (45–72)	47 (40–50)	50 (38–66)	− 3 (− 7–3)	− 5 (− 8–0)

*RR* Respiratory rate, *HR* heart rate, *SBP, DBP* and *MAP* systolic, diastolic and mean blood pressure respectively, *SI* stroke index, *CI* cardiac index and *TPRI* total peripheral resistance index (Indexed values are stroke volume; cardiac output and total peripheral resistance divided by body surface area), *EDI* End diastolic volume index

No significant differences between delirium and “controls” (Mann-Whitney tests)

^a^Values are medians (total range) obtained from registration during sequences of four minutes at rest and tilt to 15°, and changes from rest after tilt to 15°

^b^BP-recordings are missing in 1 patient with no delirium

#### Heart rate variability according to delirium status

At rest, all HRV indices were higher in the delirium group, but the differences were not statistically significant (Table 4). In particular, there was a tendency in the delirium group to higher values for LFabs (*p* = 0.06), SDNN (*p* = 0.06) and pNN50 (*p* = 0.06). At 15^0^ tilt, the delirious patients had a tendency to higher PSD (*p* = 0.06), lower LFnu and higher HFnu, and thus a lower LF/HF-ratio than the control group (*p* = 0.06). Delirious patients had a significantly more pronounced reduction in SDNN from baseline than the controls (*p* = 0.01). Patients with delirium also had a larger but non-significant reduction in PSD during HUT compared with the non-delirious group.

**Table 4:** Heart Rate Variability (Frequency Domain and Time Domain Measures)^a^ at Rest and During 15° HUT by diagnosis

Characteristic	Rest		15° tilt		Change after 15° tilt	
	Delirium, *n* = 5	No delirium, *n* = 9	Delirium, *n* = 5	No delirium, *n* = 9	Delirium, *n* = 5	No delirium, *n* = 9
Frequency Domain Measures						
PSD RRI	2400	174	6213^†^	141	− 202	13
	(350–30,075)	(16–20,659)	(148–22,462)	(46–19,083)	(−7612–8015)	(− 1576–439)
LFnuRRI	21	29	23^††^	39	2	7
	(11–62)	(17–57)	(9–65)	(25–62)	(− 28–4)	(−14–16)
HFnuRRI	79	71	77^††^	61	−2	−7
	(39–89)	(43–83)	(35–91)	(38–75)	(− 4–28)	(− 16–14)
LFabsRRI (ms^2^)	419^†^	27	854	29	−44	12
	(87–11,813)	(5–5986)	(27–11,579)	(10–5943)	(−234–860)	(− 69–163)
HFabsRRI (ms^2^)	1804	88	5267^†^	39	1	4
	(86–7371)	(8–14,150)	(87–9161)	(12–11,365)	(− 1150–7071)	(− 2785–130)
LF/HF ratio	0.3	0.4	0.3^†^	0.5	0	0.1
	(0.1–1.6)	(0.2–1.3)	(0.1–1.9)	(0.3–1.4)	(− 0.7–0.3)	(− 0.5–0.8)
Time Domain Measures						
SDNN (ms)	51^†^	25	36	22	−15 ^b^	0
	(27–227)	(8–69)	(17–174)	(13–63)	(− 54 - -5)	(− 12–16)
pNN50 (%)	11^†^	1	9	2	−1	0
	(3–68)	(0–36)	(0–86)	(0–20)	(− 10–18)	(− 16–16)
RMSSD (ms)	36	20	30	30	−9	8
	(15–151)	(7–75)	(27–33)	(19–43)	(− 120–19)	(−44–23)

LFnuRRI and HFnuRRI = low frequency and high frequency power in normalized units; LF or HF/(Total Power-VFL(very low frequency) × 100; LFabsRRI and HFabsRRI = power in high frequency and low frequency range in absolute values, respectively; LF/HF = ratio low frequency power/high frequency power; SDNN = Standard Deviation of all NN intervals; pNN50 = NN50 count divided by the total number of all NN intervals; NN50 = number of interval differences of successive NN intervals greater than 50 ms; RMSSD = Root of the Mean of the Sum of Squares of Differences between adjacent NN intervals; NN intervals = normal-to-normal intervals; BRS = Baroreceptor reflex sensibility

^†^*p* = 0.06, Mann-Whitney test

^††^*p* = 0.08, compared with controls

^a^ Values are medians and total range obtained from registration during sequences of four minutes at rest, and after tilt to 15°. Change after 15° tilt is compared to resting values

^b^*p* = 0.01, Mann-Whitney test

## Discussion

In this study, we explored possible differences in autonomic nervous activity between geriatric patients with and without delirium. Even within this small sample, there are some notable findings:

Firstly, there were no significant differences between patients with and without delirium with respect to the regulation of conventional cardiovascular variables, either at rest or during HUT. However, there was a non-significant trend for both HR and BP values to increase more during HUT in the delirious group. One possible explanation for this difference in MAP values is the existence of different central set-points of MAP between the groups. If patients in the delirium group have a higher MAP set-point, the HR and TPRI will increase more in these patients to reach this set-point during HUT [32].

Secondly, the resting values of HRV variables, both in frequency and time domains, indicate that the patients with delirium had a stronger autonomic modulation of the sinus node. At rest, patients with delirium tend to have higher HRV indices, in particular indices indicating parasympathetic control (such as HFabs, pNN50 and RMSSD), but sympathetic modulation might also be greater, as LFabs is a result of both sympathetic and parasympathetic activity. This results in higher values of PSD and SDNN (total variability) in the group with delirium. During tilt, the variability tends to fall in the delirium group (indicated by a larger reduction in PSD and SDNN) compared to the no delirium group.

Autonomic modulation will usually increase during head-up tilt. In contrast, the patients with delirium displayed a fall in many indices, resulting in a lower total variability in the delirium group. There was also no increase in the LF/HF-ratio (increased sympathovagal balance) in patients with delirium, as is normally seen during orthostatic stress. These results are difficult to interpret. One possible explanation for the lack of variability increase during tilt in the delirious patients might be that they are already using their maximal capacity for autonomic modulation at rest. They have already been brought to the fringe of their buffering capacity, and a further challenge (such as HUT) brings them “outside” the range of their homeostatic control systems, and thus variability does not increase as expected [32, 33].

Generally, high modulation is a sign of a healthy and robust regulatory system that can compensate adequately for external changes, to maintain normal homeostasis [33]. The decrease in ANS activity/modulation at tilt might indicate a less robust regulatory system/homeostatic mechanism in patients with delirium.

The patients in the delirium group had a lower functional level before hospitalisation (NEADL), as well as higher inflammatory markers (CRP and leukocytes) than the control group. This is to be expected, as older age, pre-existing cognitive impairment and functional decline are well-known predisposing factors, and infection is a common precipitating factor for delirium [1]. Frailty, cognitive impairment and infection have been previously shown to be negatively associated with HRV [6–11]. We would thus expect lower HRV in the delirium group than in the controls. However, our findings appear to indicate the opposite.

We are aware of two previous studies investigating HRV in patients with delirium [12, 13]. Zaal et al. analysed HRV measured in the frequency domain in 25 ICU patients (13 with delirium) and did not find any significant differences in HFnu and LF/HF-ratio. However, the direction of their results was such that mean HFnu was higher in patients with delirium (67 vs 57), particularly in the hypoactive state, while the LF/HF-ratio was lower in delirium. The direction of the changes was thus the same as in the present study. In addition, a recent study of HRV in ICU patients found that delirium was associated with high SDNN and RMSSD [13]. These results are also in line with those of our study.

Strengths of this study are that we have explored HRV in both the frequency domain and time domain, using a well-defined, standardized protocol including a tilt test. Our results (Table 2) indicate that the modified HUT to 15 degrees gave physiologically plausible results, even though the orthostatic challenge was small. There are also limitations to this work that should be noted. Our study is underpowered and too small to draw firm conclusions; the study is thus exploratory in nature. Many indices from the ANS were examined and, in principle, the issue of multiple comparisons should have been addressed. However, the sample size is too small to do so. The investigators who assessed the data were not blind to the patients’ delirium status. Due to multimorbidity, some patients in the ward were not eligible for the study, especially because of atrial fibrillation and the use of beta-blockers. Further, it is not clear if the HUT could be undertaken in hyperactive delirium. This may impair the generalizability of our findings.

## Conclusions

This explorative pilot study on autonomic cardiovascular control in delirium, suggests that there could be differences in HRV that should be further explored. A head-up tilt test is feasible in older medical patients, also in patients with hypoactive and mixed delirium.

## Abbreviations

ANS: Autonomic nervous system
BP: Blood pressure
BSA: Body surface area
CI: Cardiac index
CO: Cardiac output
EDV: End-diastolic volume
HF (nu): High frequency (normalized units)
HR: Heart rate
HRV: Heart rate variability
HUT: Head-up tilt
ICG: Impedance cardiography
LF (nu): Low frequency (normalized units)
MAP: Mean arterial blood pressure
pNN50: The proportion of successive RR-intervals that differ by more than 50 ms
PSD: Power spectral density
RMSSD: Square root of the mean square differences of successive RR-intervals
SDNN: Standard deviation of RR-intervals
SI: Stroke index
SV: Stroke volume
TFM: Task Force Monitor
TPR: Total peripheral resistance
TPRI: Total peripheral resistance index
VLF: Very low frequency
